# New therapeutic option for treatment-resistant depression using transcranial pulse stimulation: real-world data from a case series

**DOI:** 10.1192/j.eurpsy.2025.555

**Published:** 2025-08-26

**Authors:** A. R. Günes, M. Beglau, M. Köhne, U. Sprick

**Affiliations:** 1 Center of Neurostimulation; 2 Alexius/ Josef Clinic, Neuss, Germany

## Abstract

**Introduction:**

As a result of crises such as the Covid-19 pandemic, depressive symptoms are increasing in the European population (Arias-de la Torre *et al.* Lancet PH 2023; 8: e889-98), with depression being one of the most common illnesses anyway and are even at the highest level ever measured in some countries, like in Germany (Mauz *et al.* Front PH 2023; 11: 106). Various treatment options are available, depending on the severity and individual preference of patients, but treatment-resistant depression in particular can pose major challenges for patients and clinicians. Transcranial pulse stimulation (TPS), an alternative NIBS method based on shock waves, has been available for several years and is CE-certified for the treatment of Alzheimer’s dementia (Chen *et al.* CNS Nsc 2023; 00:1-10). It enables, for the first time, a precise and non-invasive modulation of subcortical brain regions where previously surgical intervention was necessary (Legon *et al.* Hum. Brain Mapp 2018; 39:1995-2006).

**Objectives:**

The aim of this case series is to examine safety and effectiveness of TPS in patients with treatment-resistant depression.

**Methods:**

In the present study, 5 patients (gender ratio female/male 3:2) who met the criteria for treatment-resistant depression underwent a total of 6 treatments within 14 days (each session with 6000 pulses, energy level 0.25 mJ/mm² and frequency 4 Hz). With the help of neuro-navigation using individual MRI scans, pulses were applied bilaterally in the frontal and parietal lobe as well as the precuneus area (Figure 1). Additionally the shell region of the nucleus accumbens was targeted with 300 pulses on both sides due to its involvement in the pathophysiology of depression. Written consent and the approval of an ethics committee were obtained for all patients. Before, during and immediately after the TPS intervention, the antidepressant medication of all patients remained unchanged. The Beck Depression Inventory (BDI-II) was used to assess the severity of depressive symptoms before the first and after the last treatment session.

**Results:**

Apart from a temporary feeling of pressure in the area of the temples in one patient, none of the patients experienced any side effects during and after the treatment. All patients showed a reduction in the BDI-II total score in the pre-post comparison: the mean value decreased from 41 to 26 (Figure 2).

**Image 1:**

**Figure:**
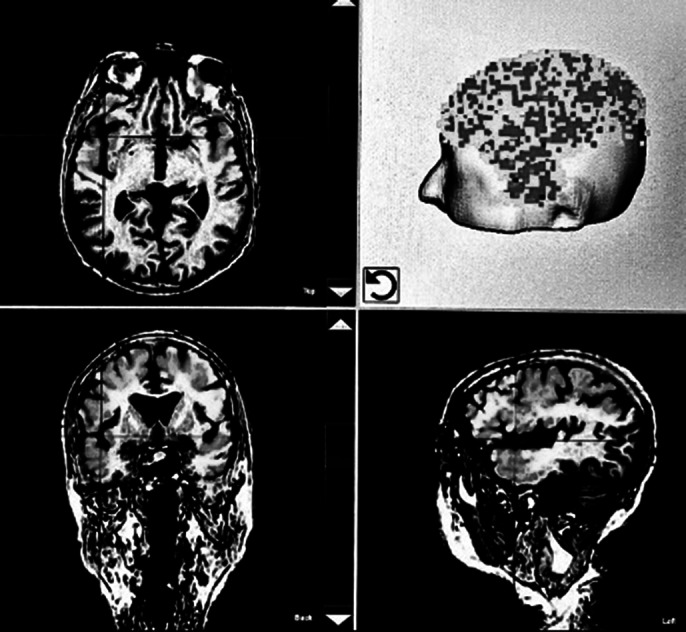

**Image 2:**

**Figure:**
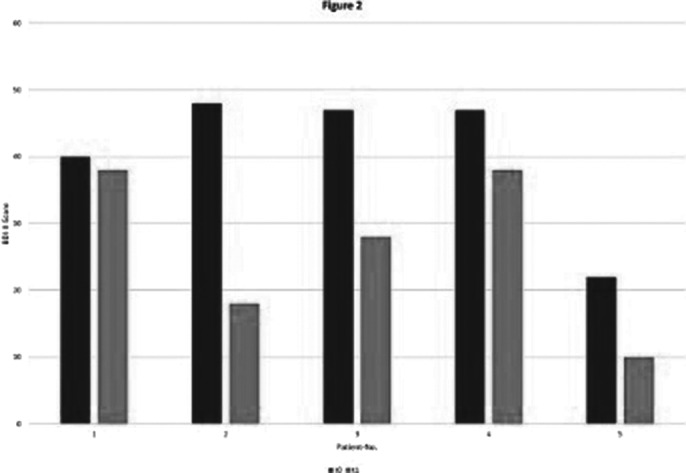

**Conclusions:**

The results of this case series show that a significant improvement in depressive symptoms is possible using TPS as a potentially well-tolerated treatment option, even in cases of treatment-resistant depression. Sham-controlled studies with large numbers of patients are required to prove its long-term effectiveness and safety.

**Disclosure of Interest:**

None Declared

